# MXene-Based Membranes for Selective Ion Separation

**DOI:** 10.3390/membranes16040146

**Published:** 2026-04-13

**Authors:** Zhiyan Zeng, Lixin Song, Li Ding, Haihui Wang

**Affiliations:** 1 School of Materials Science and Engineering, Shenyang University of Chemical Technology, Shenyang 110142, China; zengzhiyan1205@163.com; 2 Beijing Key Laboratory for Membrane Materials and Engineering, Department of Chemical Engineering, Tsinghua University, Beijing 100084, China; cehhwang@tsinghua.edu.cn

**Keywords:** MXene membrane, ion separation, two-dimensional lamellar channels, ion-sieving mechanisms, water treatment

## Abstract

Two-dimensional (2D) MXene membranes have emerged as a focal platform for ionic separations owing to their exceptional mechanical flexibility, intrinsic hydrophilicity, abundant surface terminations, and high electrical conductivity. This review summarizes recent advances in MXene-based membranes, with an emphasis on structural engineering strategies and their translation to ion-separation applications. We first outline representative fabrication routes for MXene membranes. We then discuss how separation mechanisms can be understood and deliberately tuned across four key scenarios: monovalent/monovalent ion separations, monovalent/multivalent ion separations, anion/cation separations, and heavy-metal ion separations. Finally, we highlight outstanding challenges and future opportunities, aiming to provide actionable guidance for the rational design and scalable manufacturing of high-performance MXene membranes for ionic separations.

## 1. Introduction

Water scarcity and environmental pollution remain among the most pressing global challenges of the twenty-first century [1]. A wide range of human activities—including industrial wastewater discharge, management of hypersaline brines from seawater desalination, mineral extraction, and nuclear-waste treatment—generate aqueous streams with complex ionic compositions [2,3]. Such streams may contain strategic ions (e.g., Li^+^ and K^+^) alongside highly toxic heavy-metal ions (e.g., Pb^2+^, Cd^2+^, and Hg^2+^). Achieving precise, selective separation of target ions and enabling their valorization and recovery are therefore central to environmental remediation and constitute a key scientific problem underpinning the circular economy and sustainable development [4]. Membrane-based separations have advanced rapidly over the past decades, becoming a cornerstone technology for water treatment and resource recovery because of their low energy consumption, phase-change-free operation, operational simplicity, and environmental compatibility [5]. However, conventional polymeric membranes are constrained by the well-known selectivity–permeability trade-off: enlarging pore size to enhance water flux often compromises ion rejection, whereas achieving high selectivity typically requires sacrificing permeance. This fundamental limitation largely stems from the intrinsically disordered pore architectures of polymeric membranes, which impede precise control over ion-transport pathways [6].

The emergence of 2D lamellar membranes offers a promising route to transcend this bottleneck [7,8]. In such membranes, stacked 2D nanosheets create well-defined, slit-like nanochannels in which ion transport predominantly occurs within the interlayer confined space [8,9,10,11]. This distinctive architecture enables ion sieving beyond a single-parameter, size-exclusion paradigm; instead, separation can be governed synergistically by dehydration effects induced by spatial confinement, electrostatic (Donnan) exclusion, and specific ion–channel interactions or recognition. Among the broad family of 2D materials, MXenes stand out because of their unique physicochemical attributes, making them an attractive platform for constructing high-performance ion-separation membranes [12,13,14]. MXenes are a class of 2D transition-metal carbides, nitrides, or carbonitrides, first reported in 2011, and are typically denoted by the general formula M_n+1_X_n_T_x_ (n = 1–3), where M represents an early transition metal (e.g., Ti, V, and Mo), X is carbon and/or nitrogen, and T_x_ refers to surface terminations such as –OH, –O, and –F [15]. MXenes are commonly produced by selectively etching their MAX-phase precursors—layered ternary ceramics—where removal of the A-layer element (typically Al or Si) yields an accordion-like multilayered MXene, which can be further delaminated to obtain few-layer or even monolayer nanosheets [16,17,18,19].

Compared with other 2D materials, MXenes offer several advantages for ion separation [20]. Their intrinsically hydrophilic and negatively charged surfaces promote rapid water transport while facilitating electrostatic exclusion of anions and/or multivalent cations [21]. Their interlayer spacing can be flexibly regulated through ion intercalation [22], molecular crosslinking [23], or external-field modulation [24], enabling precise control of nanochannel dimensions. In addition, their abundant surface terminations provide versatile sites for chemical functionalization, allowing the introduction of ion-recognition ligands such as crown ethers [25] and ethylene diamine tetraacetic acid (EDTA) [26,27]. MXenes also possess attractive photothermal and electrochemical properties, which broaden their utility in emerging separation technologies such as solar-driven desalination and capacitive deionization [28,29]. Compared with graphene oxide (GO), MXenes generally offer higher electrical conductivity, richer surface chemistry, and greater potential for external-field-responsive transport regulation, although GO benefits from more mature solution processability and has been more extensively studied in lamellar separation systems [9,18,19]. Compared with MoS_2_, MXenes exhibit superior hydrophilicity and more versatile interfacial chemistry, which are advantageous for regulating ion transport and selectivity [20]. However, MXenes also face important challenges, particularly oxidative degradation, restacking, and long-term structural instability in aqueous environments. Thus, their key advantage lies not in universally outperforming other 2D materials but in combining tunable interlayer spacing, chemically active surfaces, intrinsic conductivity, and multifunctionality within a single membrane platform.

**Figure 1:**
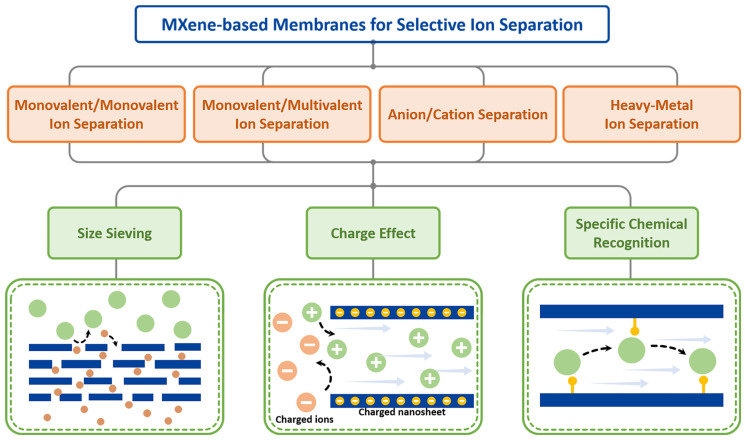
Mechanisms of ion sieving in MXene membranes and a summary of their applications in ion separation.

In recent years, MXene-membrane-enabled ion separations have advanced substantially. The field has evolved from early demonstrations dominated by simple size-based sieving to more sophisticated and programmable control enabled by intercalation engineering [18], surface functionalization [19], liquid-crystal-assisted shear alignment [20], and the coupling of external fields [21]. These strategies have expanded MXene membranes from monovalent/multivalent ion separations [22] to more challenging same-valence ion discrimination [23] and even to the precise regulation of directional anion/cation transport [24]. In this review, we systematically summarize the fabrication methods and structure-tuning strategies of MXene membranes. We then highlight progress across four representative ion-separation systems—monovalent/monovalent, monovalent/multivalent, anion/cation, and heavy-metal ion separations—dissecting the governing mechanisms in each case and, on this basis, outlining key opportunities and future directions for the field (Figure 1).

## 2. Preparation of MXene Membranes

### 2.1. Synthesis of MXene Nanosheets

Producing high-quality MXene nanosheets with large lateral size and minimal defects is a prerequisite for realizing their intrinsic properties and advancing downstream applications. At present, synthetic strategies for MXene nanosheets can be broadly classified into two categories: “top-down” and “bottom-up” approaches. Among them, top-down routes based on wet-chemical etching are the most mature and remain the dominant methods for both laboratory studies and scale-up production [25].

The core principle of top-down synthesis is to use layered ternary MAX phases as precursors and selectively remove the more weakly bonded “A” layers, thereby disassembling densely packed three-dimensional solids into two-dimensional MXene nanosheets. A representative and widely used method is hydrofluoric acid (HF) etching [15,16]. In this process, MAX-phase powders are immersed in an aqueous HF solution at relatively high concentration; the strong etching capability of HF dissolves the A layer (e.g., Al), yielding accordion-like multilayer MXene (m-MXene). Subsequently, organic intercalants such as dimethyl sulfoxide (DMSO), together with sonication, are employed to delaminate the multilayers into single-layer or few-layer nanosheets. This method is straightforward and highly efficient, and it has served as a primary route for the exploration and preparation of many MXene materials. However, the extreme toxicity and corrosiveness of HF pose severe risks to operators and the environment. In addition, the vigorous reaction can introduce structural defects and often limits the attainable lateral size of the nanosheets. To mitigate the safety concerns associated with HF, milder in situ etching protocols have been developed, typically using mixed solutions of a strong acid (e.g., HCl) and a fluoride salt (e.g., LiF) [26]. HF is generated in situ through the reaction between HCl and LiF, enabling etching of the MAX phase. Meanwhile, cations produced during etching (e.g., Li^+^) can spontaneously intercalate into the MXene interlayers, substantially weakening interlayer interactions. After etching, simple agitation or manual shaking—without additional intercalants or harsh sonication—is often sufficient to obtain a high-yield colloidal dispersion of large-area, high-quality, low-defect single-layer MXene nanosheets. Membranes fabricated from such “clay-like” MXene dispersions typically exhibit improved mechanical properties and electrical conductivity, making this approach one of the most widely adopted routes for preparing high-performance Ti_3_C_2_T_x_ MXene. Nevertheless, it still relies on strongly acidic conditions, and its applicability is somewhat narrower than that of direct HF etching, showing limited effectiveness for certain MAX phases that are intrinsically difficult to etch [17].

Beyond these mainstream wet-etching approaches, other top-down strategies—such as hydrothermal/solvothermal etching [27] and electrochemical etching [28]—have been explored to develop greener and more efficient routes. However, they have not yet become predominant, largely due to issues such as low yield and/or limited generality. In contrast, bottom-up methods, exemplified by chemical vapor deposition (CVD), directly grow large-area, high-quality, structurally well-defined MXene films (e.g., Mo_2_C) from atomic or molecular precursors (such as metallic Mo and methane) at elevated temperatures [29]. CVD-grown films typically contain very few defects, exhibit high crystallinity, and can reach lateral dimensions of tens of micrometers or more, offering distinct advantages for interrogating intrinsic properties and constructing high-performance electronic devices. However, CVD requires complex instrumentation, incurs high costs, and generally suffers from very low throughput. Moreover, only a limited number of MXenes (e.g., Mo_2_C) have been successfully synthesized by CVD to date, and the compositional versatility achievable by simply varying MAX precursors in etching-based routes is difficult to replicate. Consequently, CVD remains significantly constrained for large-scale applications and broad materials exploration [30]. Overall, selecting an appropriate MXene synthesis route requires balancing safety, yield, materials quality, cost, and the target properties required for the intended application.

### 2.2. Assembly Strategies for MXene Membranes

Assembling two-dimensional MXene nanosheets into macroscopic membranes is a critical step toward translating nanoscale properties into practical, device-relevant functionalities [31]. This process is far more than simple stacking; it requires precise control over nanosheet alignment, interlayer spacing, and the overall microstructure of the laminate [25]. To this end, a range of assembly strategies has been developed, including vacuum-assisted filtration (VAF) [32], layer-by-layer (LbL) assembly [33], spin- [34] and drop-casting [35], and electrochemical deposition [36].

Vacuum-assisted filtration remains the most widely used laboratory method for fabricating MXene membranes [32]. In a typical procedure, a dilute MXene colloidal dispersion is filtered through a porous membrane under reduced pressure, where solvent removal drives the ordered deposition of nanosheets to form a densely stacked film. VAF is operationally simple and rapid, and the membrane thickness can be precisely tuned by adjusting the MXene loading. Membranes prepared by VAF typically exhibit highly oriented lamellar architectures and compact nanosheet packing, which confer excellent mechanical flexibility as well as favorable electronic and ionic transport, underpinning strong performance in ion separation [18] and as electrodes for energy storage [37]. Nonetheless, VAF has intrinsic limitations. First, the filtration process is self-limiting, making it challenging to fabricate ultrathin, large-area, and uniform free-standing membranes. Second, the relatively slow film-forming process can lead to in-plane misalignment and pronounced restacking, which substantially reduces ion-accessible surface area and constrains performance in applications that demand high surface utilization (e.g., high-rate supercapacitors).

Layer-by-layer assembly offers a more refined route to microstructural control [33]. In this approach, a substrate is alternately immersed in solutions bearing oppositely charged species, enabling sequential deposition of MXene nanosheets and complementary functional materials via electrostatic adsorption to construct highly ordered composite films. The key advantage of LbL lies in its nanometer-scale control over thickness and composition, facilitating molecular-level homogeneity and uniform mixing of constituents—features that are particularly valuable for engineering multifunctional composite membranes with well-defined architectures and tunable properties. This level of control, however, comes at the expense of throughput: repeated rinsing and drying steps are typically required after each deposition cycle, rendering the overall process time-consuming. Consequently, LbL is better suited for fundamental studies and applications demanding stringent structural precision rather than for rapid, large-scale production of thick membranes.

Beyond these methods, a variety of solution-processing routes have also been explored. Spin coating [33] and spray coating [34] are among the simplest and fastest, using centrifugal spreading or solvent evaporation to form films on flat substrates. These approaches are compatible with existing micro-/nanofabrication workflows and are convenient for rapid preparation of films for basic characterization or small devices, but they often suffer from limited control over thickness and uniformity and are generally unsuitable for producing large-area, free-standing membranes. Solution casting, in which an MXene dispersion is poured into a mold and allowed to evaporate, is straightforward but typically slow; moreover, film uniformity is often compromised by the coffee-ring effect [38]. To mitigate restacking in conventional lamellar films, template-assisted methods [39] have been developed, where sacrificial templates are introduced to create ordered macropores or vertically aligned architectures, thereby markedly improving ion-transport kinetics within the membrane—albeit at the cost of increased process complexity. Electrochemical deposition, by contrast, exploits electric-field-driven migration of charged MXene nanosheets toward an electrode substrate, enabling rapid film growth. The microstructure can be tuned by controlling the applied potential/current and deposition time, making this strategy particularly attractive for directly coating active materials onto complex-shaped current collectors for high-performance energy-storage electrodes, although it requires adequate dispersion stability and a suitable electrochemical window [36]. Overall, each assembly strategy represents a distinct trade-off among throughput, structural precision, microstructural control, and resulting performance; the optimal choice, therefore, depends on the target application and processing constraints.

## 3. Ion Separations

### 3.1. Monovalent/Monovalent Ion Separations

Precise separation of monovalent ion pairs (e.g., Na^+^/K^+^, Na^+^/Li^+^, and Cl^−^/Br^−^) represents one of the most challenging frontiers in ion-separation science. Such capability is central to diverse, high-impact applications, including lithium extraction from salt-lake brines [40], removal of radioactive cesium from nuclear wastewater [41], maintenance of Na^+^/K^+^ balance in hemodialysis [42], and purification of specific electrolytes in biomedicine and pharmaceutical manufacturing [43]. The fundamental difficulty arises because the target ions not only carry identical charge states but also possess very similar hydrated radii, rendering conventional membrane materials that rely primarily on size-based sieving ineffective for high-fidelity recognition and separation. Consequently, achieving monovalent/monovalent ion separation hinges on the ability to precisely regulate ion-transport behavior at the sub-nanometer scale.

**Figure 2:**
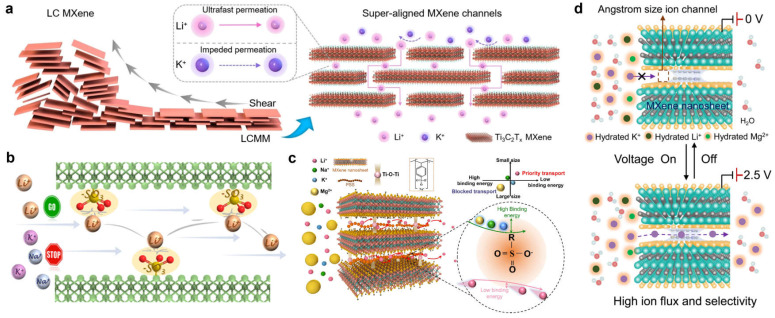
MXene-based membranes for monovalent/monovalent ion separation. (**a**) A lamellar MXene membrane featuring super-aligned nanochannels [20]. (**b**) Schematic of an MXene composite membrane with sulfonic acid groups intercalated between the layers [19]. (**c**) Structure of a swelling-resistant antilamellar MXene@PSS composite membrane [45]. (**d**) A voltage-gated MXene membrane that, in conjunction with ionic charge, enables tunable and selective ion transport [21]. Panels (**a**–**d**) are adapted with permission.

Monovalent/monovalent ion discrimination hinges on a synergistic interplay between (i) dehydration barriers imposed by precisely defined size confinement and (ii) deliberate regulation of the physicochemical environment inside sub-nanometer channels. Notably, current MXene-membrane engineering strategies remain debated with respect to which factor is truly dominant. In 2023, Kang et al. [44] constructed noncovalently modified sub-nanometer channels by forming a multivalent hydrogen-bonding network between ethanol molecules and surface terminations within Ti_3_C_2_T_x_ laminates. Without disrupting the lamellar framework, this approach leveraged the coupled effects of size sieving and dehydration-energy barriers, boosting the H^+^/Na^+^ selectivity to 12 and maintaining stable performance over 17 days. This work emphasized the predominant role of dehydration-barrier differences arising from size confinement in governing ion selectivity. In the same year, our group [20] fabricated lamellar membranes with super-aligned nanochannels by shearing a liquid-crystalline MXene dispersion, yielding uniform two-dimensional channels of ~6 Å (Figure 2a). In diffusion permeation experiments, the resulting membranes exhibited a Li^+^ permeation rate of up to 0.35 mol m^−2^ h^−1^, together with markedly enhanced ideal selectivities for Li^+^/Na^+^, Li^+^/K^+^, and Li^+^/Rb^+^ (45, 49, and 59, respectively)—selectivities that are superior to those of the vast majority of membrane materials. Theoretical analysis indicated that hydrated Li^+^ undergoes partial dehydration under confinement, leading to the smallest effective diameter, and it also shows the weakest adsorption on MXene surfaces (adsorption energy of −3.082 eV), thereby minimizing migration resistance; in contrast, other ions experience stronger adsorption and steric hindrance, which suppresses their transport. Nevertheless, the stringent requirements on feedstock quality and processing conditions may limit the scalability and practical manufacturability of this strategy.

By contrast, MXene separation membranes that exploit chemical recognition by functional molecules or groups toward specific ions have emerged as a particularly promising direction. Tong et al. [19] introduced a sulfonated polyelectrolyte, PSS bearing –SO_3_H groups, into MXene interlayers, which not only avoided interlayer contraction but instead expanded the spacing to 2.33 nm while simultaneously imparting a high density of negative charges. Taking advantage of the slightly weaker binding of Li^+^ to –SO_3_^−^ (−0.220 eV) compared with Na^+^ (−0.223 eV) and K^+^ (−0.229 eV), the membrane enabled preferential Li^+^ transport, achieving Li^+^/Na^+^ and Li^+^/K^+^ selectivities of 2.5 and 3.2, respectively—attributed to the specific affinity of sulfonate moieties toward Li^+^ (Figure 2b). Building on chemical-recognition mechanisms, our group has developed a series of functionalized MXene membrane systems. In 2021, we constructed MXene@PSS composite membranes in which self-crosslinking suppressed swelling while –SO_3_^−^ sites provided Li^+^-selective recognition, establishing a dual synergistic mechanism of “size confinement plus chemical recognition” that increased the Li^+^/Na^+^ and Li^+^/K^+^ selectivities to approximately 16 and 13, respectively (Figure 2c) [45]. More recently, we leveraged the preferential affinity of crown ethers for K^+^ to design an asymmetric bilayer membrane composed of a crown-ether-functionalized recognition layer and a pristine MXene reinforcement layer [46]. This architecture enabled accurate K^+^ recognition together with rapid transport, markedly amplifying the K^+^/Na^+^ selectivity (5–9) and thereby substantiating both the feasibility and the advantages of specific-recognition mechanisms for monovalent-ion separations.

Wang et al. [21] engineered a conductive, polydopamine (PDA)-confined MXene lamellar membrane, in which the effective transport channels in the hydrated state were constricted to ~4.6 Å. They further proposed a “voltage-gating plus ion-charge co-mediation” strategy to enable controllable and selective separation of monovalent cations. At an applied voltage of 2.5 V, the selectivities for K^+^/Li^+^, K^+^/Na^+^, and Na^+^/Li^+^ reached approximately 16, 3, and 5, respectively. Notably, upon introducing Mg^2+^ into the K^+^/Li^+^ system, the K^+^/Li^+^ selectivity further increased to roughly 41 while the K^+^ flux remained as high as ~73 m mol m^−2^ h^−1^. Mechanistically, a negative potential enhances interfacial polarization at the membrane surface, promotes dehydration of K^+^, and lowers the associated transport barrier (Figure 2d). The addition of Mg^2+^ is proposed to restructure the electrical double layer at the channel entrance, thereby strengthening the preferential enrichment and migration of K^+^ relative to Li^+^. The key innovation of this work lies in coupling voltage gating with charge-environment modulation induced by competitive ions, synergistically integrated with size sieving and dehydration-barrier effects, to achieve both high selectivity and tunable transport. Nonetheless, the demonstrated systems remain relatively model in nature, and the sensitivity of performance to operating voltage, pH, and ionic strength under practical conditions warrants further evaluation.

### 3.2. Monovalent/Multivalent Ion Separations

Monovalent/multivalent ion separations (e.g., Na^+^/Mg^2+^ and Cl^−^/SO_4_^2−^) are central to processes such as seawater desalination, hard-water softening, and food processing [3,4]. Unlike monovalent/monovalent ion discrimination, the most salient difference between the ions in this scenario is their valence state; accordingly, charge-based exclusion (e.g., Donnan exclusion) can play a dominant role.

**Figure 3:**
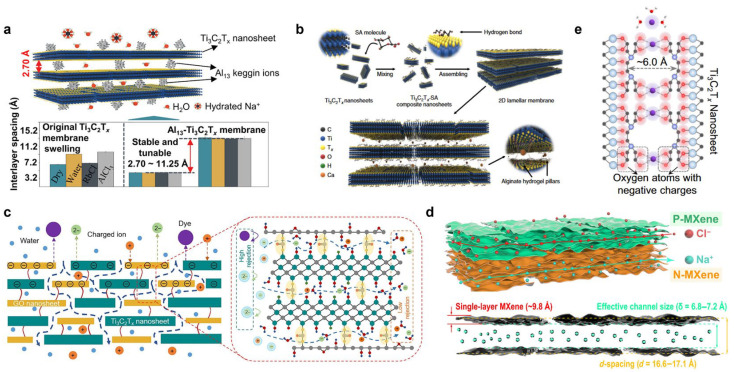
MXene-based membranes for mono-/multivalent ion separation. (**a**) An Al_13_-Ti_3_C_2_T_x_ lamellar membrane achieving ion separation through precise control of its interlayer spacing [47]. (**b**) A Ti_3_C_2_T_x_ MXene membrane with its interlayer distance stabilized at 7.4 ± 0.2 Å by an alginate hydrogel pillar [48]. (**c**) A 2D GMU hybrid membrane with tunable interlayer spacing, fabricated by crosslinking graphene oxide and MXene nanosheets with urea [49]. (**d**) Schematic illustration of a Janus NP-MXene membrane exhibiting bidirectional ion selectivity and its interlayer channels [24]. (**e**) Diagram of the negatively charged oxygen-rich nanochannels within an MLM-EDTA membrane, featuring an interlayer spacing of 6.0 Å [50]. Panels (**a**–**e**) are adapted with permission.

Zhu et al. [47] intercalated Keggin-type Al_13_ polycations into Ti_3_C_2_T_x_ MXene laminates to construct swelling-resistant two-dimensional lamellar membranes via electrostatic interactions. By tuning the Al_13_ content and the aging time, they achieved ångström-level control over the interlayer structure, with an interlayer-spacing modulation of 2.7–11.2 Å. A membrane with a narrower spacing (11.5 Å) exhibited a markedly enhanced Na^+^/Al^3+^ selectivity of nearly 173, whereas a more open laminate (20.0 Å) substantially increased the permeation of monovalent cations and outperformed conventional membranes in H^+^/Fe^2+^ separation (Figure 3a). This strategy predominantly leverages dehydration effects, rendering the separation performance highly dependent on differences in ionic hydration free energies. However, multivalent ions can in some cases exhibit smaller hydrated radii than their monovalent counterparts—for example, hydrated Mg^2+^ is smaller than hydrated Na^+^. This implies that, if one relies solely on size-based sieving, multivalent ions may even transport faster. Therefore, effectively harnessing charge effects to overcome potential interference from size sieving is a defining requirement for monovalent/multivalent separations, fundamentally distinguishing this regime from same-valence ion separations.

Wang et al. [48] stabilized the interlayer spacing of Ti_3_C_2_T_x_ MXene laminates using alginate hydrogel pillars, thereby precisely fixing the nanochannel diameter at 7.4 ± 0.2 Å (Figure 3b). This system relies primarily on dehydration-energy barriers of hydrated ions to achieve ion sieving, delivering a Na^+^/Al^3+^ selectivity as high as about 53. In acid-recovery applications, the H^+^/Fe^2+^ selectivity was improved by approximately 800-fold relative to commercial membranes. Moreover, because the membrane surface carries a high density of negative charges, surface-charge effects dominate ion transport under angstrom-scale confinement, enabling efficient rejection of Na_2_SO_4_. Yu et al. [49] cross-linked graphene oxide and MXene nanosheets with urea to construct a two-dimensional composite membrane with tunable interlayer spacing (the GMU membrane), achieving a Cl^−^/SO_4_^2−^ selectivity of up to nearly 12. These results further corroborate the synergistic contributions of size sieving, ion dehydration, and Donnan exclusion in monovalent/multivalent ion-separation systems (Figure 3c).

Building on these advances, Xu et al. [50] introduced EDTA molecules to construct biomimetic sub-nanometer channels in two-dimensional lamellar membranes. EDTA not only stabilized the channel size at approximately 6.0 Å, synergistically integrating size sieving with dehydration effects, but also provided abundant negatively charged oxygen atoms with tunable charge density and enabled precise recognition of Mg^2+^ (Figure 3e). As a result, the membrane achieved a K^+^/Mg^2+^ selectivity as high as roughly 121 in mixed-salt solutions. Extending this concept, Qian et al. [24] functionalized opposite sides of MXene with EDTA and poly-diallyldimethylammonium chloride (PDDA), respectively, to construct a Janus NP-MXene membrane featuring bidirectional ion permselectivity (Figure 3d). This bidirectional transport architecture enabled simultaneous separation of Na^+^/Mg^2+^ and Cl^−^/SO_4_^2−^, delivering selectivities of approximately 110 and 91, respectively.

Overall, monovalent/multivalent ion separations in MXene membranes are governed primarily by charge-based exclusion, with size sieving and specific coordination acting cooperatively to sharpen selectivity. Future efforts should focus on improving long-term operational stability. Multivalent ions, particularly Ca^2+^ and Mg^2+^, readily form precipitates on membrane surfaces or within interlayers or bind irreversibly to functional groups, leading to fouling and performance decay. Accordingly, developing antifouling surfaces, reversibly addressable recognition sites, and effective cleaning protocols will be crucial for translating MXene membranes toward practical deployment.

### 3.3. Anion/Cation Separations

Achieving selective ion transport in mixed salt solutions (e.g., Na^+^/Cl^−^ and Mg^2+^/SO_4_^2−^), where the membrane regulates the relative transport rates of anions and cations rather than completely separating them into distinct ionic solutions. Selective ion transport between anions and cations is central to applications such as osmotic energy conversion [51], acid/base recovery [3], and capacitive deionization [28]. A defining distinction of this regime, relative to separations between like-charged ions, is that the driving force is governed less by differences in size, dehydration barriers, or specific affinity and more by the membrane’s intrinsic charge characteristics in concert with externally applied fields. These fields can include concentration gradients, applied voltage, and light illumination, which together enable programmable regulation of ion transport and permselectivity.

**Figure 4:**
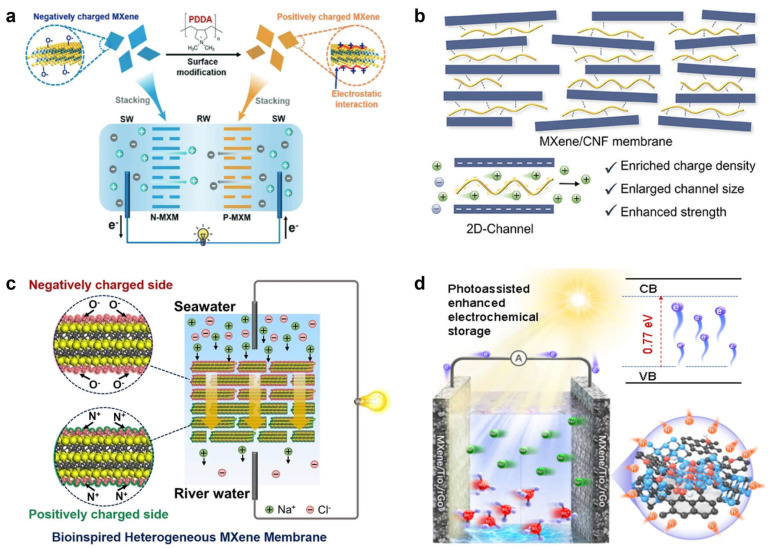
MXene-based membranes for anion/cation separation. (**a**) Oppositely charged Ti_3_C_2_T_x_ MXene membranes with 2D nanofluidic channels designed for osmotic energy harvesting [52]. (**b**) Schematic illustration of a robust MXene nanosheet/cotton nanofiber composite membrane and its internal nanochannel structure [53]. (**c**) A bio-inspired Ti_3_C_2_T_x_ MXene-based ionic diode membrane for high-efficiency osmotic energy conversion [54]. (**d**) Light-enhanced, laser-architected MXene composite for use in supercapacitor applications [55]. Panels (**a**–**d**) are adapted with permission.

In 2020, our group [52] functionalized pristine, negatively charged Ti_3_C_2_T_x_ MXene nanosheets with PDDA, reversing the surface potential from −41 mV to +48 mV. Using layer-by-layer assembly, we constructed lamellar membranes with opposite charge polarities. In the negatively charged N-MXM, the nanochannels electrostatically attract Na^+^ while excluding Cl^−^, resulting in pronounced cation selectivity (t_+_ = 0.96). By contrast, the positively charged P-MXM selectively transports Cl^−^ (t_+_ = 0.10). Integrating these two membranes into a reverse electrodialysis system enabled osmotic-energy harvesting under a salinity gradient (Figure 4a), delivering a power density of up to almost 5 W/m^2^ and an energy-conversion efficiency of approximately 44%. In 2025, we further introduced cotton nanofibers (CNFs) into MXene laminates [53], creating MXene/CNF composite membranes that combine high mechanical strength with excellent ion selectivity. Incorporation of CNFs increased the space-charge density within the membrane and enlarged the two-dimensional channel size, rendering the electrical double-layer thickness comparable to the channel dimension and thereby strengthening surface-charge-governed ion transport (Figure 4b). Consequently, Na^+^ preferentially permeates, whereas Cl^−^ is hindered; this asymmetric ion distribution generates a diffusion current and a membrane potential, enabling efficient osmotic energy conversion. Under a 50-fold salinity gradient, the membrane achieved a cation transference number of 0.90, an energy-conversion efficiency of nearly 29%, and a power density of approximately 10 W/m^2^, outperforming most reported two-dimensional membrane materials. This charge-regulation strategy—rooted in Donnan effects and electrical-double-layer-mediated ion selectivity—offers a broadly applicable and practically promising route for deploying MXene membranes in salinity-gradient energy conversion.

Inspired by the asymmetric ion channels of electric eels, our group [54] assembled a heterogeneous membrane composed of positively and negatively charged MXene nanosheets through surface-charge engineering and asymmetric structural design, enabling osmotic energy conversion in the Na^+^/Cl^−^ system. Under voltage bias, this membrane establishes ion-enrichment and ion-depletion zones, which markedly suppress concentration polarization (Figure 4c). As a result, it delivered a power density as high as roughly 18 W/m^2^ and an energy-conversion efficiency of about 39% under a 500-fold salinity gradient, demonstrating outstanding anion/cation regulation and separation performance. This strategy leverages both the high surface charge density and the readily tunable surface chemistry of MXenes while using an applied voltage to actively drive efficient anion/cation separation.

Beyond externally applied voltages, Yuan et al. [55] shifted the controlling field to a built-in electric field within a heterojunction. Using femtosecond-laser processing, they fabricated a biomimetic flower-like MXene–TiO_2_–reduced graphene oxide (LfMT) heterojunction composite for photoenhanced microsupercapacitors (Figure 4d). The laser-generated TiO_2_ serves as a photosensitizer to harvest photons, while MXene and rGO provide efficient charge-transport pathways, together enabling effective separation of photogenerated carriers and rapid adsorption/transport of ions (H^+^/SO_4_^2−^). Under illumination, the capacitance increased by 228%, reaching an areal capacitance of approximately 718 mF cm^−2^ and a volumetric capacitance as high as about 2591 F cm^−3^. The corresponding energy density and power density reached approximately 0.5 Wh cm^−3^ and 320 W cm^−3^, respectively, setting a new benchmark for comparable devices. This “field-internalized” modulation strategy offers a new paradigm for anion/cation separation-type systems and appears broadly extendable.

Overall, anion/cation separation is governed by the synergistic coupling between electrostatic interactions and electric-field regulation. Unlike purely passive diffusion, such systems typically require external energy inputs—such as salinity gradients, applied voltages, or light illumination—to drive separation. Current efforts increasingly focus on improving the stability and cycling durability of MXene-based membranes under field operation, for example, mitigating layered-structure collapse or loss of surface terminations induced by repeated charging/discharging. In parallel, designing free-standing MXene membranes that simultaneously deliver high ion-exchange capacity and robust mechanical strength remains an important and underexplored direction.

### 3.4. Heavy-Metal Ion Separations

Heavy-metal contamination by ions such as Pb^2+^, Hg^2+^, and Cd^2+^ poses severe risks to ecosystems and human health because of their high toxicity and persistence [56]. Compared with the ion-separation scenarios discussed above, heavy-metal ion separation exhibits three defining characteristics. First, heavy metals often occur at low or trace concentrations, requiring separation materials with exceptionally high affinity and adsorption capacity. Second, heavy metals can coexist in multiple valence states and/or complexed forms; for example, Cr(III) and Cr(VI) differ dramatically in toxicity, meaning that separation must often be coupled with valence-state control and speciation transformation. Third, the practical objective extends beyond “separation” to emphasize adsorption and desorption, namely, complete removal from aqueous environments followed by safe handling or disposal. The use of MXene membranes for heavy-metal separation primarily leverages abundant surface terminations as adsorption sites, together with interlayer confinement that enables effective capture of target ions [57].

**Figure 5:**
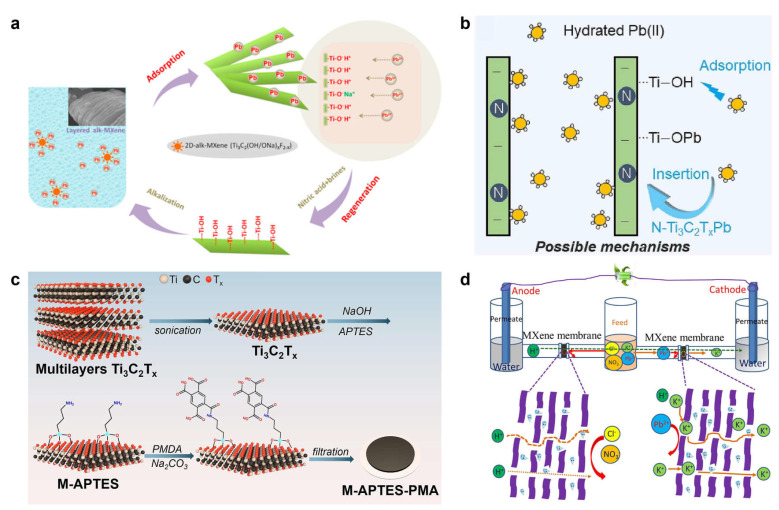
MXene-based membranes for heavy metal ion separation. (**a**) Schematic depicting the highly efficient and selective adsorption of Pb^2+^ ions by an alkalized-intercalated 2D MXene [57]. (**b**) Proposed mechanism for enhanced Pb^2+^ removal using a nitrogen-doped MXene membrane [59]. (**c**) Fabrication of a multifunctional MXene membrane via precise pyromellitic acid grafting for the effective removal of heavy metal ions [58]. (**d**) Voltage-enhanced ion sieving and lead rejection in a thermally crosslinked 2D MXene membrane [60]. Panels (**a**–**d**) are adapted with permission.

Current strategies largely focus on functionalizing MXenes to strengthen their binding toward specific heavy-metal ions. In 2014, Peng et al. [57] alkalized HF-delaminated MXene to convert surface -F terminations into activated Ti-OH/ONa groups (Figure 5a). These activated Ti-O^−^ sites serve as the primary adsorption centers for heavy-metal ions: Pb^2+^ binds strongly to form inner-sphere complexes, whereas Ca^2+^ and Mg^2+^ are less competitive because their high hydration energies hinder dehydration and coordination. This material reached adsorption equilibrium within 120 s, delivered an adsorption capacity of 140 mg g^−1^, and exhibited a treatment capacity of 4500 kg water per kg material. The residual Pb^2+^ concentration in the effluent was below 2 μg L^−1^, outperforming the WHO guideline value (10 μg L^−1^). Long et al. [58] covalently grafted carboxyl-rich pyromellitic acid (PMA) onto MXene membranes via a controlled surface-grafting strategy (Figure 5c). Under gravity-driven operation, the resulting membrane achieved removal efficiencies above 99.2% for Cu^2+^, Zn^2+^, Ni^2+^, and Cd^2+^ at an initial concentration of 10 mg L^−1^, primarily because the grafted carboxyl groups act as chemisorption sites that capture heavy-metal ions through coordination interactions. Wang et al. [59] synthesized nitrogen-doped Ti_3_C_2_T_x_ MXene (N-Ti_3_C_2_T_x_) via a hydrothermal route, significantly expanding the interlayer spacing to 1.24 nm and increasing the abundance of surface Ti-OH groups. Under an applied voltage of 1.5 V, the N-Ti_3_C_2_T_x_ cathode achieved a Pb^2+^ removal efficiency as high as 99.5%, markedly outperforming pristine MXene (84.4%). The underlying mechanism involves an electric-field-driven, insertion-type pseudocapacitive reaction of Pb^2+^, coupled with the formation of stable inner-sphere complexes with Ti-OH groups (Figure 5b). Benefiting from this combined mechanism, the Pb^2+^ removal efficiency reached 99.5% in mixed electrolytes containing Na^+^, whereas Na^+^ removal was only 15.2%. Even in the presence of competing Cu^2+^ and Ni^2+^ ions, the selectivity toward Pb^2+^ remained as high as 97.9%. After five cycles, the removal efficiency was maintained at 97.0%. This study innovatively integrates pseudocapacitive storage with surface complexation, opening a new route for selective electrochemical removal of trace-level heavy-metal ions.

Xia et al. [51] exploited the photothermal effect of MXene membranes to simultaneously enhance wastewater treatment and osmotic power generation under illumination. Focusing on the separation of Na^+^ from heavy-metal ions, they found that light-induced heating increased the Na^+^ permeation rate by 429% to almost 63 mol m^−2^ h^−1^. In contrast, permeation of heavy-metal ions (e.g., Pb^2+^ and Cu^2+^) was suppressed, yielding a maximum Na^+^/Pb^2+^ selectivity of 2050. Molecular dynamics simulations attributed this behavior to “multi-ion interactions” within angstrom-confined channels: the temperature-enhanced Na^+^ flux generates strong electrostatic repulsion against Pb^2+^ attempting to approach or enter the nanochannels. This repulsion—beyond simple Donnan exclusion—effectively pushes heavy-metal ions away from the channel entrances and/or impedes their forward migration within the channels, leading to decreased rather than increased permeation.

Fan et al. [60] fabricated two-dimensional MXene membranes via thermal crosslinking and achieved effective K^+^/Pb^2+^ sieving together with highly efficient Pb^2+^ rejection under an applied voltage (Figure 5d). During drying, dehydration and crosslinking of hydroxyl groups regulated the interlayer spacing to 6.7–6.92 Å, positioned between the hydrated diameters of K^+^ (6.62 Å) and Pb^2+^ (8.02 Å). Under an applied voltage, hydrated Pb^2+^ was rejected with an efficiency of up to 99%, whereas hydrated K^+^ permeated readily. For a 365 nm-thick MXene membrane, a separation factor of 78 for K^+^/Pb^2+^ was achieved at 16.5 V. This work highlights the potential of size sieving synergistically coupled with external electric fields to achieve high-efficiency heavy-metal ion separation, providing new design principles for electrically driven membrane processes in wastewater treatment and resource recovery.

Overall, heavy-metal ion separation by MXene-based membranes is governed by the synergy of adsorption, coordination, and size sieving. Distinct from ion separations aimed at enabling selective passage, heavy-metal removal places greater emphasis on rejection and adsorption. Accordingly, adsorption capacity, binding affinity, and long-term operational stability are the key metrics for evaluating performance.

## 4. Conclusions

Two-dimensional MXene membranes, enabled by their intrinsically hydrophilic surfaces, highly tunable interlayer nanochannels, and abundant sites for chemical modification, have demonstrated strong potential for ion separations that can outperform conventional polymeric materials. This review systematically surveys progress spanning the controllable synthesis of MXene nanosheets and multiscale membrane-assembly strategies, with particular emphasis on advances across four representative ion-separation regimes. Collectively, recent studies reveal that ion-sieving performance in MXene membranes has evolved from a framework dominated by size-based exclusion to one governed by multiple synergistic mechanisms, primarily including charge effects (Donnan exclusion and electrical-double-layer-mediated transport) and specific chemical recognition. Together, these mechanistic insights have deepened our understanding of ion transport in the confined spaces of two-dimensional MXene laminates and have established diverse, mechanistically grounded pathways to overcome the classic selectivity–permeability trade-off that limits traditional membrane materials.

However, several challenges still need to be addressed before MXene membranes can move toward practical implementation. These challenges mainly concern the intrinsic susceptibility of MXene membranes to oxidative degradation and the difficulty of large-scale manufacturing, with the former having a particularly significant impact on their long-term stability under realistic operating conditions. One critical issue is the tendency of MXene membranes to undergo oxidation and structural degradation over time. Owing to the intrinsic reactivity of their exposed transition-metal surfaces, MXenes are readily oxidized upon contact with water or oxygen. Such oxidation may lead to aggravated membrane swelling, loss of precise control over interlayer spacing, and declines in ion selectivity and permeance, ultimately compromising membrane performance during prolonged operation [13,14,15]. To address this issue, researchers have explored strategies such as self-crosslinking [61] and ion [47] or molecular intercalation [45] to stabilize the interlayer spacing within the membrane. At the same time, efforts have also been devoted to improving the sedimentation tendency of MXene nanosheets and the instability of MXene membranes by precisely regulating the surface terminal groups of MXene nanosheets [62] or by adopting edge-sealing strategies [63]. Although various approaches have been developed to improve the long-term stability of MXene membranes, there remains a lack of systematic, simple, and broadly applicable regulation strategies. Another key challenge facing MXene membranes is the difficulty of scaling up fabrication to produce large-area, high-quality membranes. At present, it remains difficult to assemble MXene nanosheets into large-area, freestanding membranes using methods such as VAF [32] or LBL [33]. Conventional large-scale membrane fabrication techniques include doctor blading [34], spray coating [35], and roll-to-roll processing. However, these methods are subject to inherent limitations, such as poor in-plane alignment, nanosheet restacking, thickness nonuniformity over large areas, and difficulties in controlling the coating slurry. Therefore, developing scalable fabrication technologies capable of producing uniform, defect-free membranes at low cost remains a critical challenge. Further exploration and optimization of approaches such as roll-to-roll processing, continuous assembly, and alternative template-assisted methods will be required to meet the demand for large-area MXene membranes.

Therefore, future research should focus on the following directions. First, simple yet generalizable approaches for precisely tuning the interlayer environment are urgently needed to address the persistent challenge that many functionalization strategies struggle to simultaneously deliver high efficiency and long-term stability. Second, the structural robustness, antifouling capability, and regenerability of MXene membranes under realistic operating conditions require more systematic and standardized evaluation. Third, developing continuous, scalable fabrication processes for large-area membranes will be essential to bridge laboratory demonstrations and practical deployment. Overall, MXene membranes provide an attractive platform for engineering next-generation, high-performance ion-separation materials. With sustained mechanistic interrogation and process innovation, this field is well-positioned to contribute to global challenges spanning water security, energy, and environmental health.

## Data Availability

No new data were created or analyzed in this study. Data sharing is not applicable to this article.
